# Types of surgical patients enrolled in enhanced recovery after surgery (ERAS) programs in the USA

**DOI:** 10.1186/s13741-021-00185-5

**Published:** 2021-04-27

**Authors:** Sunitha M. Singh, Asha Liverpool, Jamie L. Romeiser, Julie Thacker, Tong J. Gan, Elliott Bennett-Guerrero

**Affiliations:** 1 Department of Anesthesiology, Stony Brook University Medical Center, 101 Nicholls Road, Health Science Center, L-4, 060, Stony Brook, NY 11794-8480 USA; 2 Department of Surgery, Duke University Medical Center, 10 Duke Medicine Circle, Durham, NC 27710-1000 USA

**Keywords:** Enhanced recovery, ERAS, ERP

## Abstract

**Background:**

Enhanced Recovery After Surgery (ERAS) programs have gained traction across US hospitals in the past two decades. Initially implemented for elective colorectal surgical procedures, ERAS has expanded to a variety of surgical service lines. There is little information regarding the extent to which various surgical service lines use ERAS.

**Methods:**

A survey was performed to describe the prevalence of ERAS programs across surgical service lines in the USA. The survey had questions regarding the number of ERAS programs, operating rooms (ORs) and presence of anesthesia and/or surgery residency program at an institution. The survey was administered electronically to members of the American Society for Enhanced Recovery (ASER) and manually to participants at the 2018 Perioperative Quality and Enhanced Recovery Conference in San Francisco, CA.

**Results:**

Responses were received from 88 unique institutions. The most commonly reported surgical service lines were colorectal (87%), gynecology (51%), orthopedic (49%), surgical oncology (39%), and urology (35%). A significant positive association was observed between the number of ORs and the number ERAS programs (Spearman’s Rho 0.5, *p*<0.0001). Furthermore, institutions that reported an anesthesia and/or surgery residency program had more ERAS programs (mean 5.0 ± 3.2) compared to those that did not (mean 2.0 ± 2.0) (Wilcoxon rank sum *p*< 0.001).

**Conclusions:**

ERAS has expanded to a large extent outside of the colorectal surgery service line with increases notable in orthopedic surgery, obstetric/gynecology, surgical oncology, and urology procedures. Institutions with a higher number of ORs and the presence of an anesthesia and/or surgery residency program are associated with an increased number of ERAS programs.

## Background

Enhanced Recovery After Surgery (ERAS) programs are evidence-based, multidisciplinary, surgical care pathways developed to expedite recovery and improve patient outcomes after elective surgery (Desiderdio et al. 2018; McConnell et al. 2018; Brown et al. 2018; Ban et al. 2019). ERAS, comprised of several specific, evidence-based protocols, allows for the standardization of surgical patient care preoperatively, intraoperatively, and postoperatively. Their use within the colorectal service line has been well-studied and, as a result, ERAS programs have expanded into additional surgical specialties (e.g., thoracic, cardiac, gynecology, and orthopedic). Yet, little is known about the prevalence of ERAS among various surgical services in the United States (US).

Therefore, we conducted a nationwide survey to characterize the prevalence of ERAS programs across different surgical service lines. By identifying the types of elective surgical patients enrolled in an ERAS pathway and the types of institutions implementing ERAS pathways, we aim to inform the ERAS community on the extent to which ERAS programs are used across the USA and to encourage more research into its use across various surgical service lines.

## Methods

### Data collection

Institutional review board (IRB) approval was obtained from Stony Brook University Medical Center IRB. The 9-question survey asked about the types of ERAS pathways, the number of operating rooms (ORs), and the presence of an anesthesia and/or surgery residency program at the respondent’s main hospital. The survey was designed, tested, and distributed electronically using the Qualtrics® (Provo, UT) online software to members of the American Society for Enhanced Recovery (ASER) between September and November 2018. The survey was also circulated to participants at the October 2018 Perioperative Quality and Enhanced Recovery Conference in San Francisco, CA.

### Statistical analysis

Analysis was performed at the institutional level. To not over represent, i.e., double-count, institutions in the study, only the first completed survey was included for analysis from each institution. This response was selected first by completion rate, then by time/date submitted. If completion rates for the surveys were both 100% (fully completed survey), the first survey response received was used to represent the institution. Duplicate responses from institutions, surveys from unknown institutions, and non-US site responses were excluded from the analysis. Associations between the number of ERAS programs, the number of ORs, and the presence or absence of an anesthesia and/or surgery residency program, which served as surrogates for hospital size and academic status, were examined using Spearman’s rank and Wilcoxon rank-sum tests. Survey data related to carbohydrate beverage use will be published in a separate manuscript.

## Results

Overall, 148 completed surveys were received, representing 88 unique hospitals. Respondents identified themselves as anesthesiologist (44.3%), ERAS coordinator (19.3%), surgeon (14.8%), nurse (10.2%), certified registered nurse anesthetist (3.4%), other (3.4%), nurse practitioner (2.3%), and dietitian (2.3%).

The majority of hospitals reported having an adult colorectal ERAS program (88.6%), followed by gynecology (51.1%), orthopedic (48.9%), surgical oncology (38.6%), and urology (35.2%). The least reported programs were thoracic (15.9%), ear-nose-throat [ENT] (14.8%), vascular (11.4%), cardiac (9.1%), and craniotomy (1.1%). Of the 88 hospitals, 4.6% reported not having any ERAS program. In total, there were fourteen programs reported, including “other,” as noted in Table 1.

**Table 1:** ERAS programs reported

Active adult ERAS programs	***n*** (%)
Colorectal	78 (88.6)
Gynecology	45 (51.1)
Orthopedic	43 (48.9)
Surgical oncology	34 (38.6)
Urology	31 (35.2)
Spine	21 (23.9)
Bariatric	20 (22.7)
Plastic	20 (22.7)
Obstetric	18 (20.5)
Thoracic	14 (15.9)
ENT	13 (14.8)
Vascular	10 (11.4)
Cardiac	8 (9.1)
Other	6 (6.8)
None	4 (4.6)
Craniotomy	1 (1.1)

Associations between the number of ERAS programs and the number of ORs and the number of ERAS programs and the presence/absence of an anesthesia or surgery residency program are described in Table 2. There was a 3-fold difference in the average number of ERAS programs by number of ORs between the smallest and largest categories, 2.2 ± 1.8 (1–10 ORs) compared to 6.4 ± 3.6 (>40 ORs). There was a significant positive association between the number of ORs and the number of ERAS programs (Spearman’s Rho 0.5, *p*<0.0001). Moreover, institutions that reported an anesthesia and/or surgery residency program had more ERAS programs (mean ± SD, 5.0 ± 3.2) compared to those that did not (mean ± SD, 2.0 ± 2.0, Wilcoxon rank sum < 0.001). Sensitivity analyses confirmed that responses were similar for electronically and manually administered surveys.

**Table 2:** Associations between the number of ERAS programs and the number of ORs and the number of ERAS programs and the presence/absence of an anesthesia or surgery residency program

**Number of operating rooms (*n*=87)**	**Number of ERAS programs**				**Spearman’s Rho**
	***N***	**Mean ± SD**	**Min**	**Max**	
1–10	26	2.6 ± 1.8	1	7	Rho = 0.5, *p*<0.001
11–20	25	4.1 ± 2.8	1	10	
21–40	20	5.0 ± 3.0	1	12	
>40	16	6.4 ± 3.6	1	13	
**Anesthesia and/or surgery residency program (*n*=86)**	**Number of ERAS programs**				**Wilcoxon rank sum**
	***N***	**Mean ± SD**	**Min**	**Max**	
No	35	2.3 ± 2.0	1	10	*p*<0.001
Yes	51	5.3 ± 3.2	1	13	

## Discussion

ERAS elements span the preoperative, intraoperative, and postoperative care phases. Standardizing these components has had a positive impact on the overall quality of recovery (e.g., decreased surgical stress, length of stay, and reduced complications) (Ban et al. 2019).

In accordance with anecdotal reports, our survey shows within institutions that are likely to implement ERAS pathways, many enroll colorectal patients. Within these institutions however, our survey results suggest that ERAS has expanded, and pathways are being applied to a wide variety of other surgical services lines. As such, the ERAS® Society has promulgated evidence-based recommendations for many surgical service lines to guide perioperative patient care and improve healthcare delivery and outcomes.

We observed that institutions that are larger and/or have an anesthesia or surgery residency program are more likely to have more ERAS programs. We did not assess the reason(s) for this, but can speculate that larger institutions are more likely to have resources that can be devoted to expansion of ERAS and other quality improvement efforts. Regardless of size, encouraging sustainable ERAS expansion requires institutional commitment and support to embed ERAS as a standard model of care across multiple therapeutic areas. Once a hospital expands ERAS into multiple surgical procedures, it becomes impractical to use manual data capture. Therefore, IT resources are needed to allow for automated data capture and analysis including audit of compliance with bundle elements in order to optimize outcomes and patient satisfaction.

Our study has several potential limitations. The goal was not to assess the rate of ERAS expansion within the USA or the specific elements in the different pathways, e.g., goal-directed therapy; therefore, we cannot comment on how rapidly ERAS programs are being developed and implemented nor which ERAS elements are consistently used across different surgical service lines. Though our survey was not piloted prior to distribution, it was internally tested. It was administered electronically first to ASER members and then in-person (manually) to conference attendees, possibly leading to inconsistencies in responses. Yet, a sensitivity analysis showed similar results for electronic and manual survey responses. This is not surprising as we would not expect these results to markedly differ based on how the survey was delivered. Additionally, these results may be more generalizable to clinicians who are interested in enhanced recovery, as they are more likely to have attended the ASER meeting or be an ASER member and thus be approached and willing to complete the survey.

## Conclusion

In summary, our study found that ERAS is being implemented in many different types of elective surgery. Additionally, centers with a higher number of ORs have a larger number of ERAS programs. Additional studies are needed to address the growth rate of ERAS programs within the USA, the shared elements between different ERAS pathways, and sustainable ways to support ERAS expansion across varied institutional settings.

## Data Availability

The datasets used are not available.

## Abbreviations

ASER: American Society for Enhanced Recovery
ENT: Ear, nose, throat
ERAS: Enhanced Recovery After Surgery
IRB: Institutional Review Board
OR: Operating room
SD: Standard deviation
